# A novel non-invasive murine model for rapidly testing drug activity via inhalation administration against *Mycobacterium tuberculosis*

**DOI:** 10.3389/fphar.2024.1400436

**Published:** 2025-01-03

**Authors:** Xirong Tian, Yamin Gao, Chunyu Li, Wanli Ma, Jingran Zhang, Yanan Ju, Jie Ding, Sanshan Zeng, H. M. Adnan Hameed, Htin Lin Aung, Nanshan Zhong, Gregory M. Cook, Jinxing Hu, Tianyu Zhang

**Affiliations:** ^1^ State Key Laboratory of Respiratory Disease, Joint School of Life Sciences, Guangzhou Chest Hospital, Guangzhou Institutes of Biomedicine and Health, Chinese Academy of Sciences, Guangzhou Medical University, Guangzhou, China; ^2^ Guangdong-Hong Kong-Macao Joint Laboratory of Respiratory Infectious Diseases, Guangzhou Institutes of Biomedicine and Health (GIBH), Chinese Academy of Sciences (CAS), Guangzhou, China; ^3^ University of Chinese Academy of Sciences (UCAS), Beijing, China; ^4^ China-New Zealand Joint Laboratory on Biomedicine and Health, Guangzhou Institutes of Biomedicine and Health, Chinese Academy of Sciences, Guangzhou, China; ^5^ School of Life Sciences, University of Science and Technology of China, Hefei, China; ^6^ Institute of Physical Science and Information Technology, Anhui University, Hefei, China; ^7^ Department of Microbiology and Immunology, School of Biomedical Sciences, University of Otago, Dunedin, New Zealand; ^8^ Maurice Wilkins Centre for Molecular Biodiscovery, University of Auckland, Auckland, New Zealand; ^9^ Guangzhou National Laboratory, Guangzhou, China; ^10^ Translational Research Institute, Queensland University of Technology, Brisbane, QLD, Australia

**Keywords:** inhalation administration, autoluminescence, tuberculosis, murine model, chemotherapy

## Abstract

The efficacy of many compounds against *Mycobacterium tuberculosis* is often limited when administered via conventional oral or injection routes due to suboptimal pharmacokinetic characteristics. Inhalation-based delivery methods have been investigated to achieve high local therapeutic doses in the lungs. However, previous models, typically employing wild-type *M. tuberculosis* strains, were intricate, time-consuming, labor-intensive, and with poor reproducibility. In this study, we developed an autoluminescence-based inhalation administration model to evaluate drug activity by quantifying relative light units (RLUs) emitted from live mice infected with autoluminescent *M. tuberculosis*. This novel approach offers several advantages: (1) it eliminates the need for anesthesia in mice during administration and simplifies the instrument manipulation; (2) it is cost-effective by utilizing mice instead of larger animals; (3) it shortens the time from several months to 16 or 17 days for obtaining result; (4) it is non-invasive by directly measuring the live RLUs of mice as a surrogate marker for colony-forming units for *in vivo* drug activity testing; (5) up to six mice can be administrated daily and simultaneously, even 2–3 times/day; (6) results are relatively objective and reproducible results minimizing human factors. Proof-of-concept experiments demonstrated that inhalable rifampicin, isoniazid, and ethambutol showed anti-*M. tuberculosis* activity at concentrations as low as 0.5, 0.5, and 0.625 mg/mL, respectively, as evidenced by comparing the live RLUs of mice. Furthermore, consistency between RLUs and colony-forming units of the autoluminescent *M. tuberculosis* in lungs reaffirms the reliability of RLUs as an indicator of drug efficacy, highlighting the potential of this approach for accurately assessing anti-*M. tuberculosis* activity *in vivo*. This autoluminescence-based, non-invasive inhalation model offers a substantial reduction in the time, effort, and cost required for evaluating the efficacy of screening new drugs and repurposing old drugs *in vivo* via inhalation administration.

## Introduction

*Mycobacterium tuberculosis*, primarily transmitted through respiratory droplets, is the causative agent of human tuberculosis (TB) and is responsible for the highest mortality rate attributed to a single infectious agent before the 2019 coronavirus pandemic (McQuaid et al., 2021; Shah et al., 2022). The emergence of drug-resistant *M. tuberculosis* strains presents substantial public health challenges and economic burdens (Dousa et al., 2020; Hameed et al., 2018; McGrath et al., 2014; Shoen et al., 2018). Therefore, there is an urgent need for innovative *in vivo* drugs screening and evaluation tools to identify efficacious antimicrobial agents for TB control.

Conventional *in vivo* efficacy evaluation experiments for anti-TB drugs often employ oral or various injection routes of administration (Namvarpour et al., 2019; Yu et al., 2020; Yu et al., 2021). However, these methods fail to specifically target the lungs and may induce significant toxicity in other organs during absorption, transport, and metabolism (Xu et al., 2019; Yoon et al., 2017; Zhao et al., 2023). Over several decades, inhalation therapy, delivering high local therapeutic doses to the lungs, has emerged as a preferred treatment method for respiratory diseases (Barnes et al., 2015; Garcia-Contreras et al., 2007; Gonzalez-Juarrero et al., 2012; Hickey et al., 2016; Sanchis et al., 2013). However, previous attempts to develop inhalable anti-TB drug administration models have encountered several challenges, including the time-consuming process of counting colony-forming units (CFUs), the complexities associated with anesthesia during administration, the difficulty in instrument operation, and the poor repeatability of experimental results (Garcia-Contreras et al., 2010; Verma et al., 2013).

Meanwhile, most existing drugs evaluation models mentioned above are based on wild-type *M. tuberculosis* and require several months for obtaining results, as it takes 3–6 weeks for the *M. tuberculosis* to form visible colonies on agar plates. Recently, some researchers have introduced autoluminescent *M. tuberculosis* into the process of drugs or vaccine evaluation (Yang et al., 2015; Zhang et al., 2012). The *luxAB* genes encode the enzymes that catalyze the reaction resulting in the emission of blue-green light, and *luxCDE* genes are responsible for the synthesis and recycling of the aldehyde substrate (Hakkila et al., 2002). Light production by the autoluminescent *M. tuberculosis* carrying the *luxCDABE* gene cluster has been utilized as a surrogate of CFUs or visible bacterial growth for high-throughput evaluation of antimycobacterial compounds *in vitro* and *in vivo* (Li et al., 2023; Yang et al., 2015; Zhang et al., 2012). The combination of improved inhalation delivery method and autoluminescent *M. tuberculosis* may offers a promising approach for assessing drug activities.

Here, we have developed an autoluminescence-based, inhalable, time-efficient, non-invasive, and cost-effective antimicrobial assessing model that provides a valuable tool for evaluating the *in vivo* efficacy of lead compounds, frontline anti-TB medications, and therapeutic regimens, thereby offering insights crucial for clinical practice.

## Materials and methods

### Mycobacterial strains and culture conditions

The genetically engineered autoluminescent *M. tuberculosis* H37Rv (AlRv) strain, which harbors the *luxCDABE* gene cluster within its genome, was preserved at −80°C (Yang et al., 2015). AlRv displays a consistent emission of blue-green light independently, a characteristic attributed to the enzymatic activity encoded by the *luxAB* genes. *luxCDE* genes are responsible for the continuous production of the substrate aldehyde necessary for bioluminescence. Remarkably, AlRv demonstrates drug susceptibility and growth rate comparable to that of the wild-type *M. tuberculosis* strain. The traditional quantification method of CFUs was replaced by the assessment of relative light units (RLUs), maintaining an approximate ratio of 10:1.

The AlRv was subcultured in Middlebrook 7H9 broth (Difco, Detroit, MI, United States), supplemented with 10% oleic-acid-albumin-dextrose-catalase enrichment medium (BBL, Sparks, MD, United States), 0.2% glycerol, and 0.05% Tween80 at 37°C. The AlRv culture was grown until the optical density, measured at 600 nm, reached 0.6–0.8, with a corresponding RLUs level of 5 × 10^6^/mL. The optical density was measured using a V-1000 spectrophotometer (AOE, Shanghai, China), and the RLUs were quantified using the GLOMAX 20/20 luminometer (Promega, Madison, United States).

### Antimicrobials

The inhalation delivery model for this experiment was constructed using three first-line anti-TB drugs with high solubility in water: rifampicin (RIF), isoniazid (INH), and ethambutol (EMB). All pharmaceutical drugs were purchased from Meilun (Dalian, China) with a minimum purity threshold of ≥95%. RIF and INH were solubilized in sterilized water, serving as the solvent, to attain stock concentrations of 2 mg/mL, which were subsequently diluted to 0.5 and 0.25 mg/mL, respectively. EMB was similarly dissolved into sterilized water at its original concentration of 10 mg/mL, then further diluted to 2.5 and 0.625 mg/mL. The prepared solutions of the drugs were refrigerated at 4°C until administration.

### Aerosol infection

The female BALB/c mice (Gempharmatech, Foshan, China), aged six to eight weeks, underwent an acclimatization period of five to seven days before the initiation of experimental procedures. Subsequently, all mice were exposed to 10 mL broth culture of AlRv using an inhalation exposure system (Glas-Col, Terre Haute, IN) (Yu et al., 2020; Yu et al., 2021).

### Determination of the initial bacterial burden

After infection, the mice were randomly divided into treatment and control groups, each containing six mice. Anesthesia was administered on the day treatment commenced, which corresponded to the third day post-infection in Experiment 1 and the day after infection in Experiment 2. Each mouse was placed individually into a container containing 0.5 mL of isoflurane for anesthesia induction. It took around 10 seconds for the loss of consciousness. Subsequently, the live RLUs of mice were measured by aligning their chests with the detection position of the GLOMAX 20/20 luminometer. After detection of the live RLUs, six mice were euthanized via cervical dislocation and positioned on a foam board with large head pins to secure them, ensuring their abdomens were oriented upwards. The mice were sterilized by spraying 75% ethanol and dissected to obtain the lungs. The organs were then placed into 2 mL sterilized phosphate-buffered saline, washed once with phosphate-buffered saline, and homogenized using glass tissue grinders. The lung suspensions were diluted using a 10-fold serial dilution method. Subsequently, the RLUs of 0.5 mL of undiluted and 10-fold diluted lung suspensions were measured using the GLOMAX 20/20 luminometer. Based on the detected RLUs and the ratio of CFUs to RLUs, the CFUs of the lung suspension were briefly speculated upon. The 0.5 mL properly diluted lung suspension was plated on 7H10 plates containing trimethoprim, actidione, carbenicillin, and polymyxin B to prevent contamination. All plates were then incubated in a 37°C thermostatic incubator for 4 weeks, following which the CFUs were determined.

### Chemotherapy and measurement of bacterial burden

Each cohort of six mice underwent daily administration of either 4 mL of solvent or drug solutions utilizing the Tow Systems Nose-Only Exposure Units (Tow-Int, Shanghai, China), as previously described (Fan et al., 2022; Yang et al., 2021; Zhao et al., 2023). The device of administration is illustrated in Supplementary Figure S1. Mice were subsequently secured in a holder and connected to the exposure cabinet. The drug or solvent solution was injected into the atomizer, which uses high-frequency oscillation technology through microporous screens to generate nebulized aerosols with diameters ranging from 1 to 5 μm for inhalation. The drug exposure duration for each group was approximately 20–25 min until 4 mL of the solutions had been administered.

The study consists of two main experiments, delineated as Experiment 1 and Experiment 2. The experimental protocols remained largely consistent, with the primary variations involving alterations in pharmacological dosages and the timing of medication administration subsequent to infection. The detailed designs for Experiments 1 and 2 were provided in Tables 1, 2, respectively. The live RLUs of the mice in the solvent group were continuously monitored from the day 11 for Experiment 1 (or day 13 for Experiment 2) post-infection. Measurements of RLUs for all groups were performed and the treatment stopped once the live RLUs of the solvent-treated mice exceeded 400. The duration of administration for Experiments 1 and 2 was 14 and 15 days, and the final live RLUs were recorded respectively (Tables 1, 2). Subsequently, all mice were euthanized the following day to quantify lung RLUs and CFUs using the above-mentioned method. The plates were then incubated at 37°C for 4 weeks, and then CFUs were counted.

**TABLE 1:** Scheme of experiment 1

Drug/Dosage^a^ (mg/mL)	Number of mice sacrificed at the given time points				Total
	D0^b^	D3^c^	D11-17^d^	D18^e^	
Solvent	Infection	6	Detecting chest RLUs of live mice in solvent group daily from day 11 until live RLUs exceed 400	6	12
RIF 2		Treatments initiation		6	6
INH 2				6	6
EMB 10				6	6
Total	30	6		24	30

^a^ RIF, rifampicin; INH, isoniazid; EMB, ethambutol; Solvent, sterilized water; the number after the drug represents the dosage.

^b^ D0, a total of 30 mice were infected with AlRv by aerosol.

^c^ D3, the mice were randomly divided into groups and treated daily from the day 3 to day 16. The live RLUs, the lung suspension RLUs, and CFUs, of six mice were detected on Day3 before the treatment initiation.

^d^ D17, live RLUs of all mice were detected and treatment stopped.

^e^ D18, the mice were sacrificed and the lungs were removed for detecting RLUs and CFUs.

All CFUs were counted 4 weeks after incubation at 37°C.

**TABLE 2:** Scheme of experiment 2

Drug/Dosage^a^ (mg/mL)	Number of mice sacrificed at the given time points				Total
	D0^b^	D1^c^	D13-16^d^	D17^e^	
Solvent	Infection	6	Detecting chest RLUs of live mice in solvent group daily from day 13 until live RLUs exceed 400	6	12
RIF 2		Treatments initiation		6	6
RIF 0.5				6	6
RIF 0.125				6	6
INH 2				6	6
INH 0.5				6	6
INH 0.125				6	6
EMB 10				6	6
EMB 2.5				6	6
EMB 0.625				6	6
Total	66	6		60	66

^a^ RIF, rifampicin; INH, isoniazid; EMB, ethambutol; Solvent, sterilized water; the number after the drug represents the dosage.

^b^ D0, a total of 66 mice were infected with AlRv by aerosol.

^c^ D1, the mice were randomly divided into groups and treated daily from day 1 to day 15. The live RLUs, the lung suspension RLUs, and CFUs, of six mice were detected before the treatment initiation.

^d^ D16, live RLUs of all mice were detected and treatment stopped.

^e^ D17, the mice were sacrificed, and the lungs were removed for detecting RLUs and CFUs.

All CFUs were counted 4 weeks after incubation at 37°C.

### Measurement of pharmacokinetic parameters

The CFUs data revealed that RIF at a concentration (mg/mL) of 2, INH at 0.5, and EMB at 10 demonstrated significant *in vivo* anti-mycobacterial efficacy, as evidenced by the results of Experiments 1 and 2. These three concentrations were subsequently selected for further pharmacokinetic studies.

The mice were acclimated for 5 days prior to the commencement of the experiment. Subsequently, a volume of 4 mL of each drug was administered to six mice per group for each timepoint via inhalation for a precisely defined duration of 25 min. Blood samples were collected after picking eyeballs of mice (anaesthetized by inhalation of isoflurane) at various time points: 0 min (no treatment), 5 min, 15 min, 30 min, 45 min, 1 h, 2 h, 4 h, 8 h, 16 h, and 24 h after inhalable administration. A minimum of 300 µL of blood was collected from each mouse. Subsequently, the blood samples were transferred to anticoagulant-coated tubes and gently agitated to prevent haemolysis, ensuring exposure to the anticoagulant on the inner walls of the tubes. The blood samples were then centrifuged at 4°C and 3,000 rpm for 10 min. The resulting supernatant was transferred to new centrifuge tube and stored at −80°C for subsequent analysis. The mice that had undergone blood sampling were subsequently euthanised using cervical dislocation. Lung tissues were then excised, and residual blood and tissue fluids were removed with tissue paper. Distilled water was added to lung tissue in a ratio of 1.0 g vs. 1.0 mL. The lungs were subsequently homogenized to achieve a homogeneous mixture. A mixed standard acetonitrile solution was prepared, containing 50 ng/mL of each compound: verapamil, warfarin and methylsulfonylurea. A volume of 100 μL of the plasma sample or lung suspension was added to a tube, followed by the addition of 300 μL of the acetonitrile solution containing the corresponding internal standard compound. The mixture was vortexed and shaken for 30 s, then centrifuged at 12,000 rpm at 4°C for 10 min. The supernatant was transferred and filtered through a 0.22-μm filter into a new tube, then preserved at −80°C. All samples were submitted to liquid chromatography-mass spectrometry analysis (Q Exactive Focus, Thermo Fisher) for determination of the concentrations of the drugs.

### Statistical analysis

Log-transformed data using GraphPad Prism version 8.3.0 was used to determine drug efficacy through a two-way analysis of variance, with *p* < 0.05 as statistical significance.

## Results

### Activity of a single dose of RIF, INH and EMB administered via inhalation

**FIGURE 1:**
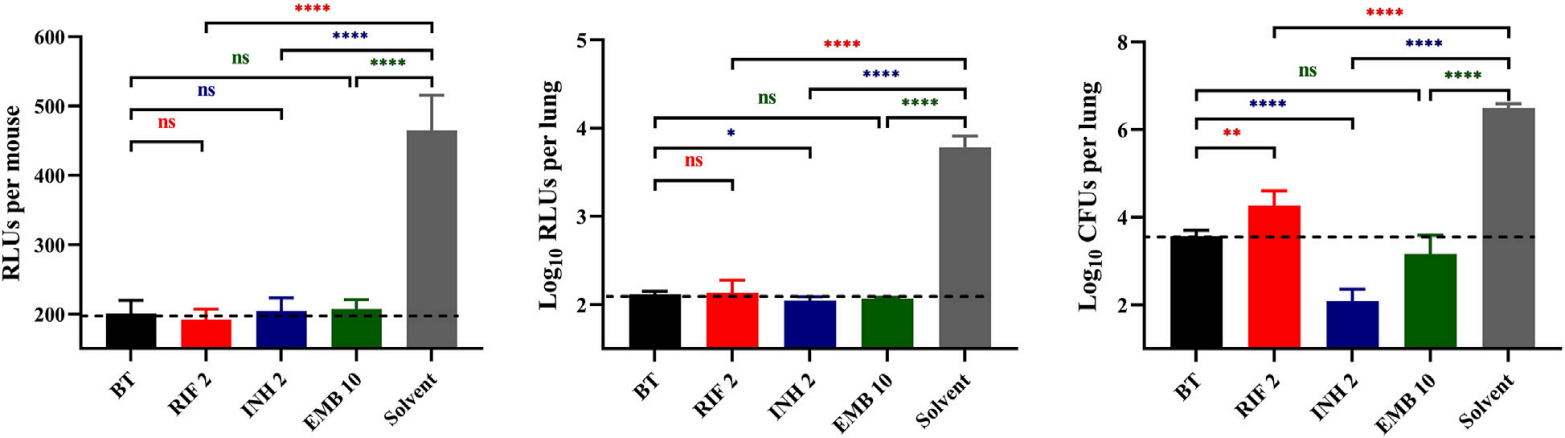
Anti-TB drug activities by inhalation administration in Experiment 1. BT, before treatment; RIF, rifampicin; INH, isoniazid; EMB, ethambutol; Solvent, sterilized water; the number after the drug represents the dosage; ^*^, *p* < 0.05; ^**^, *p* < 0.01; ^***^, *p* < 0.001; ^****^, *p* < 0.0001; ns, *p* > 0.05, no significance. The dotted lines represent the background values.

In Experiment 1, the autoluminescent *M. tuberculosis* burden in mice lungs was 3.60 ± 0.12 log_10_CFU/lung assessed on the third day post-infection, preceding the commencement of inhalable treatments (BT in Figure 1). The live RLUs of all treatment groups were significantly lower than those of the solvent group (Figure 1, *p* < 0.0001). Additionally, these live RLUs were comparable to those of the before-treatment (BT) group (Figure 1, *p* > 0.05). Furthermore, the lung RLUs of all drug-treated groups were substantially lower than those of the solvent group (Figure 1, *p* < 0. 0001). Specifically, the lung RLUs of the RIF or EMB-treated groups were nearly identical to those of the BT group (Figure 1, *p* > 0.05), while the INH-treated group exhibited slightly lower lung RLUs than the BT group (Figure 1, *p* < 0.05). Moreover, the lung CFUs of all treatment groups were markedly lower than those of the solvent group (Figure 1, *p* < 0.0001). The RIF group had higher lung CFUs, whereas the INH group had lower lung CFUs compared to the BT group (Figure 1, *p* < 0.01 and *p* < 0.0001, respectively). The lung CFUs of the EMB-treated group were almost similar to those of the BT group (Figure 1, *p* > 0.05). The data from experiment 1 indicated that the autoluminescence-based inhalation delivery method can be utilized to establish a non-invasive model for testing drugs activities *in vivo*, using the same batch of live mice within 18 days after infection. Furthermore, the bactericidal effectiveness of drugs can be further evaluated by comparing lung CFUs of the different treatment groups rather than RLUs, as lung RLUs of the BT group were close to the background values.

### Dose-dependent activity of RIF, INH and EMB administered via inhalation

**FIGURE 2:**
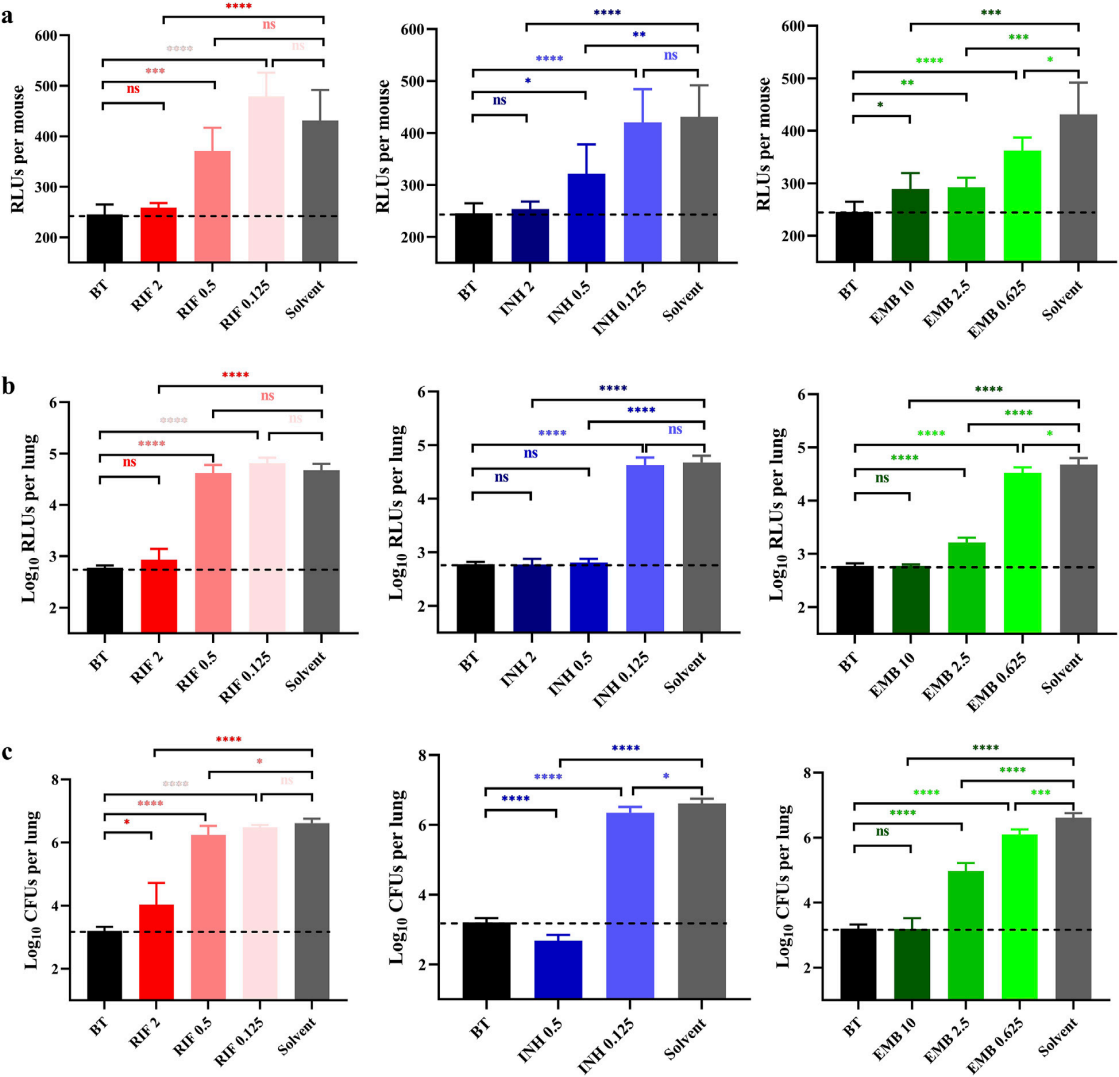
Anti-TB drug activities by inhalation administration in Experiment 2. **(A)** Light intensities of live mice. **(B)** The suspension light intensities. **(C)** The lung *Mycobacterium tuberculosis* burden. BT, before treatment; RIF, rifampicin; INH, isoniazid; EMB, ethambutol; Solvent, sterilized water; the number after the drug represents the dosage; ^*^, *p* < 0.05; ^**^, *p* < 0.01; ^***^, *p* < 0.001; ^****^, *p* < 0.0001; ns, *p* > 0.05, no significance. The dotted lines represent the background values.

Based on the results of Experiment 1, two additional dosages of each drug were added in Experiment 2. The bacterial burden in mice lungs measured on the day treatment initiated in Experiment 2 were 3.20 ± 0.11 log_10_CFU/lung (BT in Figure 2C), which approximately equivalent to those recorded in Experiment 1 (BT in Figure 1). The live RLUs of the groups treated with RIF 2, INH 2, INH 0.5, EMB 10, EMB 2.5, and EMB 0.625 mg/mL were significantly lower compared to those of the solvent group (Figure 2A, *p* < 0.0001, *p* < 0.0001, *p* < 0.01, *p* < 0.001, *p* < 0.001, and *p* < 0.05, respectively). Conversely, the live RLUs of RIF 0.5, RIF 0.125 and INH 0.125 mg/mL groups did not show significant differences from those of the solvent group (Figure 2A, *p* > 0.05). These findings were also largely supported by both the lung RLUs and CFUs (Figures 2B,C), except for the lung CFUs of the RIF at 0.5 mg/mL group, which were slightly significantly lower than those of the solvent group (Figure 2C, *p* < 0.05). In summary, these findings suggest a negative correlation between live RLUs and administered dosages, indicating that live RLUs could serve as an initial criterion for assessing the therapeutic efficacy.

The lung RLUs of the RIF 2 mg/mL group demonstrated a significant reduction compared to those in the solvent group, with a log_10_RLUs/lung difference exceeding 1.5 and even similar to that of the BT group (Figure 2B, *p* < 0.0001 and *p* > 0.05, respectively). Conversely, the lung RLUs in the RIF 0.5 and 0.125 mg/mL groups were comparable to those in the solvent group, indicating a lack of evident anti-*M. tuberculosis* activity at these dosages (Figure 2B, *p* > 0.05). The lung RLUs of the INH 2 and 0.5 mg/mL groups were significantly lower than those of the solvent group (Figure 2B, *p* < 0.0001), as all differences in log_10_RLUs/lung exceeded 1.5 and were comparable to those of the BT group (Figure 2B, *p* > 0.05). The lung RLUs of the INH 0.125 mg/mL group were similar to those of the solvent group (Figure 2B, *p* > 0.05). In comparison, the lung RLUs of the EMB 10 and 2.5 mg/mL groups exhibited a pronounced decrease compared to those of the solvent group, with log_10_RLUs/lung differences exceeding 1.5 and 1, respectively (Figure 2B, *p* < 0.0001). Meanwhile, the lung RLUs of the EMB 10 mg/mL group were equivalent to those of the BT group (Figure 2B, *p* > 0.05), whereas the lung RLUs in the EMB 0.625 mg/mL group showed less significant differences from the solvent group (Figure 2B, *p* < 0.05). In conclusion, the lung RLUs results are consistent with those obtained from the live RLUs, providing further support for the utility of detecting live RLUs in determining drug activity.

The lung CFUs in the RIF 2 mg/mL group exhibited a significant reduction compared to those in the solvent group with a difference in log_10_CFUs/lung exceeding 1.5 (Figure 2C, *p* < 0.0001). The lung CFUs in the RIF 0.5 and 0.25 mg/mL groups were approximately comparable to those in the solvent group (Figure 2C, *p* < and close to 0.05 and *p* > 0.05, respectively). Notably, no colonies were observed on the plates of the INH 2 mg/mL group, indicating possibly eradication of AlRv from the mice lungs, considering that only a fraction of the lung suspension from each mouse was plated. The CFUs of the INH 0.5 mg/mL group were also significantly lower than those of the solvent group, with a difference of log_10_CFUs/lung exceeding 1.5 and even lower than that of the BT group (Figure 2C, *p* < 0.0001). Moreover, all EMB dosages resulted in significantly reduced lung CFUs compared to the solvent control (Figure 2C, *p* < 0.0001, *p* < 0.0001, and *p* < 0.001, respectively). However, only the log_10_CFUs/lung difference between the EMB 10 mg/mL and solvent group exceeded 1.5, with the lung CFUs in the EMB 10 group being nearly identical to those in the BT group (Figure 2C, *p* > 0.05). These CFU results were consistent with the findings derived from live or lung RLUs data. Overall, based on the comprehensive experimental data presented, we have successfully established an autoluminescence-based inhalation administration murine model for evaluating drugs activities against *M. tuberculosis in vivo*.

### Pharmacokinetic properties of RIF, INH, and EMB administered via inhalation

**FIGURE 3:**
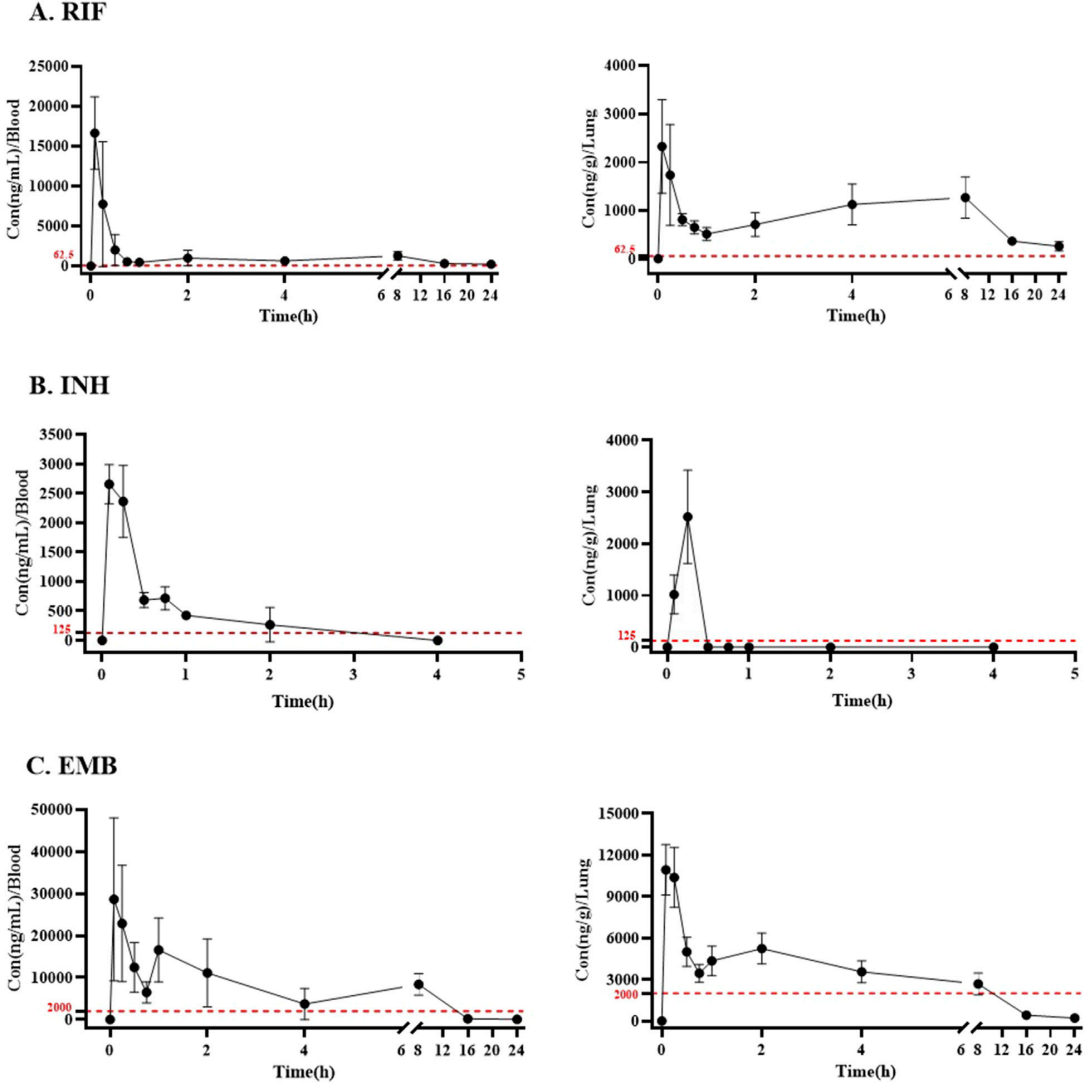
The plasma and lung concentration-time curves of **(A)** RIF, **(B)** INH, and **(C)** EMB via inhalation. Drug and dosage (mg/mL for 25 min): RIF, rifampicin (2); INH, isoniazid (0.5); EMB, ethambutol (10). The dotted red lines present the MICs of RIF, INH, and EMB against *Mycobacterium tuberculosis in vitro*.

The concentrations of RIF, INH, and EMB in plasma and lung are illustrated in Figure 3. Pharmacokinetic properties of these three drugs in plasma and lung are summarized in Table 3.

**TABLE 3:** Pharmacokinetic properties of RIF, INH, and EMB in plasma and lung via inhalation.

Drugs^a^/Parameter^b^	RIF		INH		EMB	
	Plasma	Lung	Plasma	Lung	Plasma	Lung
AUC_0-t_ (ng·h/mL)	18992.34	17474.77	1572.19	336.83	103152.20	47287.79
AUC_0-∞_ (ng·h/mL)	20966.69	20612.58	1793.18	—	103362.88	48701.18
MRT_0-∞_ (h)	9.44	12.76	0.85	—	5.11	6.38
t_1/2_ (h)	6.27	8.23	0.58	—	2.83	4.56
T_max_ (h)	0.08	0.08	0.08	0.25	0.08	0.08
C_max_ (ng/mL)	16648.48	2327.82	2655.51	2517.73	28638.14	10937.88

^a^ Drug and dosage (mg/mL for 25 min): RIF, rifampicin (2); INH, isoniazid (0.5); EMB, ethambutol (10).

^b^ AUC_0-t_, area under the concentration-time curve from zero to the maximum sampling time; AUC_0-∞_, area under the concentration-time curve from zero to infinity; MRT_0-∞_, mean residence time; t_1/2_, the half-life; T_max_, time of maximum observed plasma concentration; C_max_, maximum observed plasma concentration.

The peak concentration of RIF was observed in both plasma and lung tissues 5 min post-administration following a single inhalation dose of 2 mg/mL, with values of 16.65 ± 3.91 μg/mL and 2.33 ± 0.89 μg/g, respectively (Table 3; Figure 3A). The maximum concentration of INH was observed in both plasma and lung samples, reaching values of 2.66 ± 0.29 μg/mL and 2.52 ± 0.81 μg/g, respectively, within 5 and 15 min post-inhalation at a dose of 0.5 mg/mL (Table 3; Figure 3B). However, INH was found to be undetectable in the lungs of mice 30 min post-administration (Figure 3B). The peak concentration of EMB was reached within 5 min following inhalation administration at a dose of 10 mg/mL, in both plasma and lung (Figure 3C). The peak concentrations of EMB were 28.64 ± 16.80 μg/mL in plasma and 10.94 ± 1.63 μg/g in lung (Table 3; Figure 3C).

The area under the concentration-time curve from zero to the maximum sampling time (AUC_0-t,_), which was 4 h for INH and 24 h for RIF and EMB, was calculated to be 18.99, 1.57 and 103.15 μg h/mL in plasma, respectively (Table 3). The area under the concentration-time curve from zero to infinity (AUC_0-∞_) for RIF, INH, and EMB in plasma was 20.97, 1.79, and 103.36 μg h/mL, respectively (Table 3). The mean residence time (MRT_0-∞_) of RIF, INH, and EMB in plasma was 9.44, 0.85, and 5.11 h, respectively (Table 3). The half-life (t_1/2_) of RIF, INH, and EMB in plasma was determined to be 6.27, 0.58, and 2.83 h, respectively (Table 3).

Thirty minutes post-administration, INH concentrations in the lungs of mice were found to be nearly undetectable, thereby precluding the determination of certain pharmacokinetic parameters, including AUC_0-∞_, MRT_0-∞_, and t_1/2_. The AUC_0-t_ of INH in the lungs of mice was 0.34 μg h/mL (Table 3). The AUC_0-t,_ AUC_0-∞_, MRT_0-∞_, and t_1/2_ of RIF in the lungs of mice were 17.47 ng·h/mL, 20.61 ng·h/mL, 12.76 h, and 8.23 h, respectively (Table 3). The AUC_0-t,_ AUC_0-∞_, MRT_0-∞_, and t_1/2_ of EMB in the lungs of mice were 47.29 ng·h/mL, 48.70 ng·h/mL, 6.38 h, and 4.56 h, respectively (Table 3).

## Discussion

*M. tuberculosis* imposes a substantial burden, particularly in developing countries (Chong and Lim, 2009; Grüber, 2020). Despite ongoing efforts, the development of effective anti-*M. tuberculosis* drugs remains confronted with numerous challenges, including very slow growth rate of *M. tuberculosis* and the high cost of the biosafety facilities (McGrath et al., 2014; Olaru et al., 2015). Over the past several decades, inhalable therapy has emerged as a promising approach for treating pulmonary infections, offering improved cure rates and bacterial eradication (Riveiro et al., 2023). However, the assessment of inhalable anti-*M. tuberculosis* drugs continues to encounter challenges, including intricate manipulation (Supplementary Table S1) and the time-consuming nature of obtaining results. Recent researches have revealed that a recombinant autoluminescent strain of *M. tuberculosis* carrying *luxCDABE* gene cluster facilitates rapid, non-invasive, real-time monitoring of the RLUs in live mice during experimental chemotherapy, providing a valuable tool for assessing treatment efficacy (Yang et al., 2015; Zhang et al., 2012). Hence, we have developed a novel murine model by combining the improved inhalation drug delivery method with autoluminescent *M. tuberculosis*.

To date, four primary research reports, encompassing three inhalation administration models, have explored the detection of anti-TB drug efficacy through airway administration (Garcia-Contreras et al., 2007; Garcia-Contreras et al., 2010; Gonzalez-Juarrero et al., 2012; Verma et al., 2013). Among these studies, only two have employed murine models for their investigations. Supplementary Table S1 presents a comprehensive comparison of the advantages and disadvantages of each report relative to our study. Compared to these existing inhalation administration model, our innovative method achieves several improvements. Firstly, it is cost-effective and user-friendly, employing mice instead of larger animals. Secondly, it eliminates the need for anesthesia in mice during administration, simplifying the instrument operation. We also endeavored to utilize a published inhalation administration method using a syringe-like device in mice infected with AlRv. However, we encountered significant challenges in administering drugs daily due to the development of swollen throats in the mice (Gonzalez-Juarrero et al., 2012). However, we did observe that mice treated with INH exhibited reduced RLUs compared to both the untreated control and the same mice at the day before administration (unpublished data). Thirdly, the results obtained are relatively objective and repeatable, thereby minimizing the influence of human factor. Fourthly, the same treatment group, consisting of six mice, is administrated daily and simultaneously. We also successfully administered the same six mice by inhalation 2 or 3 times per day (data not shown). Additionally, our method is non-invasive, measuring the live RLUs of mice as a surrogate marker for CFUs for drug *in vivo* activity testing. Lastly, it reduces the time required for obtaining results from several months to 16 or 17 days.

We have evaluated the *in vivo* efficacy of three first-line TB drugs using our model, including RIF, INH, and EMB. Our model can accelerate assessment of drug activity, with results obtainable within 17 or 18 days or even shorter by detecting either live RLUs of mice or RLUs of lung suspensions. Our findings demonstrate that RIF, INH, and EMB, at the dosage of 0.5, 0.5, and 0.625 mg/mL, exhibited significant anti-*M. tuberculosis* activity *in vivo*. Notably, INH exhibits potential for complete eradication of lung bacteria at increased dosage up to 2 mg/mL in the current model. Moreover, the CFUs results corroborate the findings inferred from live RLUs, affirming the utility of live RLUs as a surrogate marker for CFUs in mice. RIF is a bactericidal anti-TB drug, yet its efficacy depends on the dosage and duration of treatment. Notably, in this study, even RIF at a concentration of 2 mg/mL failed to demonstrate bactericidal activity as evidenced by both lung suspension RLU and CFU (Figure 2B). Moreover, EMB at a concentration of 10 mg/mL exhibited superior performance compared to RIF at a concentration of 2 mg/mL (Figures 2B, C). Observations indicated that RIF might not be as effective when delivered via inhalation as it is when administered orally, which could be attributed to the specifics of the delivery route. So, it is interesting to test the contribution of anti-*M. tuberculosis* drug(s) in the regimens in curing TB by inhalation in the active TB model in the future. This innovative model holds promise for enhancing the assessment of anti-*M. tuberculosis* drug efficacy *in vivo*, thereby reducing the likelihood of overlooking potentially effective treatments and minimizing the risk of adverse effects.

Following oral administration, RIF, INH and EMB reached their highest plasma concentrations in mice at 1.33, 0.25 and 1.33 h post-administration, respectively (Grewal et al., 2018; Grosset et al., 1992; Lyons et al., 2013). When these drugs were delivered through inhalation, they rapidly attained markedly peak plasma concentrations of 16.65 ± 3.91, 2.66 ± 0.29, and 28.64 ± 16.8 μg/mL, respectively, within just 5 min post-inhalation in our study. The shortest time point we used in this study after complementation of inhalation was 5 min, and the duration of the inhalation was 25 min long. Therefore, we cannot exclude the possibility that the concentrations of drugs in the plasma may have reached their peak earlier. These findings indicate that inhalable administration can accelerate the absorption and penetration of drugs into the blood, and may also be a viable approach for treating diseases affecting other organs. The *in vitro* minimum inhibitory concentrations (MICs) of RIF, INH, and EMB against *M. tuberculosis* are 0.06, 0.13, and 2.00 μg/mL, respectively. Following inhalation administration, the plasma concentrations of RIF, INH, and EMB exceeded their respective *in vitro* MICs against *M. tuberculosis* for durations of approximately 24, 3, and 14 h, respectively. Within lung tissue, these drugs maintained their concentrations above their *in vitro* anti-*M. tuberculosis* MICs for approximately 24, 0.5, and 10 h, respectively. These findings indicate that RIF and EMB are capable of maintaining elevated concentrations in plasma and lungs for an extended period following inhalation administration, thereby exhibiting enhanced antimicrobial efficacy. However, the concentration of INH in the lung tissue notably decreases to near-zero within 30 min post-inhalation, implying a rapid clearance or metabolism of INH in the lung. This observation underscores the potential benefit of multiple daily inhalation doses to maintain an effective concentration in the lung, thereby enhancing therapeutic outcomes against pulmonary infections like TB. Overall, the pharmacokinetic profiles demonstrate that inhalation administration facilitates rapid achievement of high drug concentrations in both plasma and lung tissues, surpassing the required therapeutic thresholds and indicating a promising strategy for improving the treatment of respiratory infections caused by *M. tuberculosis*.

Our aim is to develop a rapid, simple, and cost-effective method for assessing the activity of compounds via inhalation. Primarily, this method is intended for *in vivo* drug screening via inhalation. If a compound exhibits promising activity, further investigations, such as pharmacokinetic studies, will be conducted. This explains why we did not perform pharmacokinetic studies on the selected anti-TB drugs used in this study. Establishing a chronic mouse model is time-consuming, and the light intensity is insufficient for detection in live mice due to the requirement of a low-dose infection of an autoluminescent *M. tuberculosis* strain (Zhang et al., 2012). Additionally, we did not test an active TB disease mouse model with a high *M. tuberculosis* burden. For instance, if a compound has only weak or delayed anti-TB activity (such as clofazimine, Ammerman et al., 2017; Yu et al., 2021), its effect may be overlooked in the rapid and high *M. tuberculosis* burden screening model. In such cases, the advantages of using the autoluminescent *M. tuberculosis* strain may not be evident, and questions regarding the strain may arise, such as whether the RLUs correlate well with CFUs and whether the light production could influence the drug’s effect. Therefore, if a compound shows activity in the current inhalation model, it will undergo more in-depth testing, such as pharmacokinetic study, using the wild-type *M. tuberculosis* strain in various mouse models.

The drug’s activity can be determined by comparing the lung suspension RLUs of different groups within a shorter duration than the current 17-day period (from infection to get the results), since the lung RLUs become significantly higher than the background value a few days earlier than the live RLUs do. Furthermore, drug efficacy could potentially be observed within a considerably shorter time frame in live mice if mice infected with high dose AlRv via tail vein injection route, similar to the previous studies showed that the live RLUs could be detected at the day after infection, although this may result in a higher standard deviation (Liu et al., 2019; Olaru et al., 2015). Further investigation is warranted to determine whether other anti-TB drugs or potent compounds demonstrate efficacy using our model. Additionally, exploring alternative formulations or solvents may further enhance the drug’s effectiveness of drugs beyond sterilized water. The solubility of poorly soluble and insoluble compounds can be enhanced by employing solubilizers, which facilitates the evaluation of their *in vivo* activity using this inhalation system. Meanwhile, dry powders can also be administrated via this inhalation system. However, it is necessary that these compounds are formulated into dry powders with specific particle sizes with diameters between 0.5–5 μm, and the device used in this study must be equipped with a dry powder generator.

In conclusion, our study establishes an autoluminescence-based, inhaled, and non-invasive administration murine model for evaluating anti-*M. tuberculosis* drug *in vivo* efficacy. This model holds promise for expediting the development of innovative anti-*M. tuberculosis* drugs and treatment regimens, addressing the urgent need for more effective TB therapies. Meanwhile, this improved inhalation model can also be used for developing drugs targeting other diseases and assessing the toxicity of compounds delivered by inhalation.

## Data Availability

The datasets presented in this study can be found in online repositories. The names of the repository/repositories and accession number(s) can be found in the article/Supplementary Material.
