# Supplementary material for: A novel non-invasive murine model for rapidly testing drug activity via inhalation administration against Mycobacterium tuberculosis
Source: Front Pharmacol. 2025 Jan 3;15:1400436. doi: 10.3389/fphar.2024.1400436 (PMC11739085; doi:10.3389/fphar.2024.1400436)
Supplement: Supplementary file 1 [file DataSheet1.docx]

**A Novel Non-invasive Murine Model for Rapidly Testing Drug Activity via Inhalation Administration against *Mycobacterium tuberculosis***

Xirong Tian ^1,2,3,4*^, Yamin Gao ^1,2,3,4*^, Chunyu Li ^1,2,3,4*^, Wanli Ma ^1,2,3,4^, Jingran Zhang ^1,2,3,5^, Yanan Ju ^1,2,3,5^, Jie Ding ^1,2,3,6^, Sanshan Zeng ^1,2,3,4^, H.M. Adnan Hameed ^1,2,3,4^, Htin Lin Aung ^4,8,9^, Nanshan Zhong ^7^, Gregory M. Cook ^4,8,9,10^, Jinxing Hu ^1,7#^ and Tianyu Zhang ^1,2,3,4,7#^

^1^ State Key Laboratory of Respiratory Disease, Joint School of Life Sciences, Guangzhou Institutes of Biomedicine and Health, Chinese Academy of Sciences, Guangzhou, China; Guangzhou Medical University, Guangzhou, China; Guangzhou Chest Hospital, Guangzhou, China.

^2^ Guangdong-Hong Kong-Macao Joint Laboratory of Respiratory Infectious Diseases, Guangzhou Institutes of Biomedicine and Health (GIBH), Chinese Academy of Sciences (CAS), Guangzhou 510530, China

^3^ University of Chinese Academy of Sciences (UCAS), Beijing 100049, China

^4^ China-New Zealand Joint Laboratory on Biomedicine and Health, Guangzhou Institutes of Biomedicine and Health, Chinese Academy of Sciences, Guangzhou 510530, China

^5^ School of Life Sciences, University of Science and Technology of China, Hefei 230026, China

^6^ Institute of Physical Science and Information Technology, Anhui University, Hefei, 230601, China

^7^ Guangzhou National Laboratory, Guangzhou 510005, China

^8^ Department of Microbiology and Immunology, School of Biomedical Sciences, University of Otago, Dunedin, 9054, New Zealand

^9^ Maurice Wilkins Centre for Molecular Biodiscovery, University of Auckland, Private Bag 92019, Auckland, 1042, New Zealand

^10^ Translational Research Institute, Queensland University of Technology, Brisbane, Queensland, 4102, Australia

^*^ These authors contributed equally to this work.

^#^ Corresponding author: Jinxing Hu ([hujinxing2000@163.com](mailto:hujinxing2000@163.com)) and Tianyu Zhang ([zhang_tianyu@gibh.ac.cn](mailto:zhang_tianyu@gibh.ac.cn))


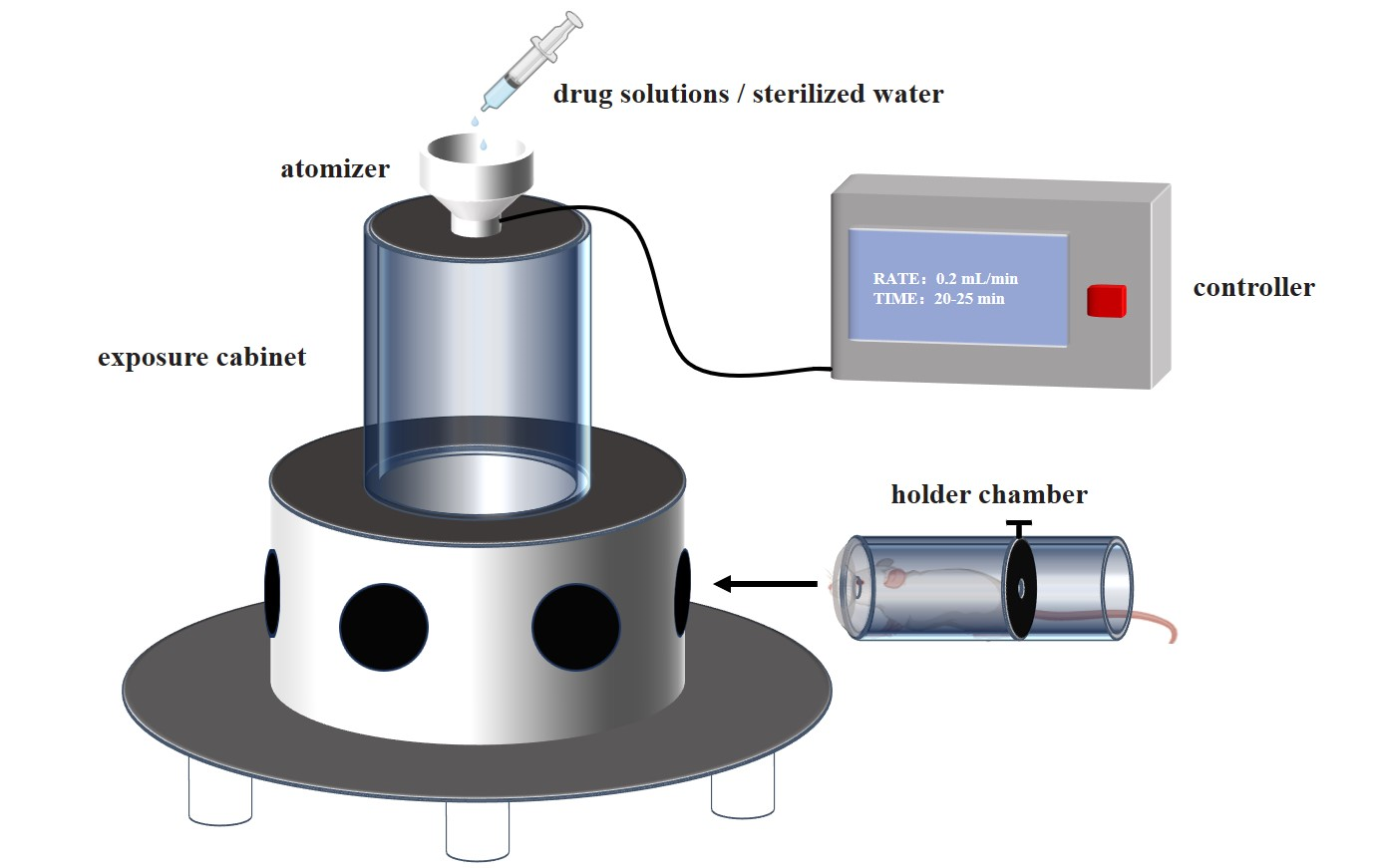


**Figure S1. Schematic diagram of inhalation administration using the Tow Systems Nose-Only Exposure Units.**

**Table S1. Summary of comparative strengths and weaknesses of the inhaled administration studies.**

| Animal | Anesthesia | Standardization  /Repeatability | No. of animals administered each time | Operability | Drug amount | Administration speed | Administration frequency | Live detection of activities | Time to get results after administration of the last dose |
| --- | --- | --- | --- | --- | --- | --- | --- | --- | --- |
| Mouse**^(1)^**^*^ | Yes | Low | 1 | Hard | Small | ~15 min | 3/week | No | 3-5 weeks |
| Guinea pig**^(2, 3)^** | - | High | 4 | Hard | Media | 30 min/low-dose or 60 min/ high-dose | daily | No | 3-5 weeks |
| Mouse**^(4)^** | - | High | 1 | Hard | Small | - | 2/week | No | 3-5 weeks |
| Mouse**^(this study)^** | No | High | 6 | Easy | Large | 20~25 min | One to several times/day | Yes | 1 day |

^*^ We also tried this method and found the throat of the mouse showed somewhat swelling after administration, so it was hard or even almost impossible to be administer the same mouse daily.

-, unclear.

**REFERENCES**

1. Gonzalez-Juarrero M, Woolhiser LK, Brooks E, DeGroote MA, Lenaerts AJ. (2012). Mouse model for efficacy testing of antituberculosis agents via intrapulmonary delivery. *Antimicrob. Agents. Chemother*. 56(7), 3957-3959.

2. Garcia-Contreras L, Fiegel J, Telko MJ, Elbert K, Hawi A, Thomas M, VerBerkmoes J, Germishuizen WA, Fourie PB, Hickey AJ, Edwards D. (2007). Inhaled large porous particles of capreomycin for treatment of tuberculosis in a guinea pig model. *Antimicrob. Agents. Chemother*. 51(8), 2830-2836.

3. Garcia-Contreras L, Sung JC, Muttil P, Padilla D, Telko M, Verberkmoes JL, Elbert KJ, Hickey AJ, Edwards DA. (2010). Dry powder PA-824 aerosols for treatment of tuberculosis in guinea pigs. *Antimicrob. Agents. Chemother*. 54(4), 1436-1442.

4. Verma RK, Germishuizen WA, Motheo MP, Agrawal AK, Singh AK, Mohan M, Gupta P, Gupta UD, Cholo M, Anderson R, Fourie PB, Misra A. (2013). Inhaled microparticles containing clofazimine are efficacious in treatment of experimental tuberculosis in mice. *Antimicrob. Agents. Chemother*. 57(2), 1050-1052.
